# Uniform Polymer Microspheres by Photoinduced Metal‐Free Atom Transfer Radical Precipitation Polymerization

**DOI:** 10.1002/marc.202400502

**Published:** 2024-11-05

**Authors:** Tugrul Cem Bicak, Huiyin Liu, Karsten Haupt, Carlo Gonzato, Jérôme Fresnais, Christine Ménager, Louis Fensterbank, Cyril Ollivier, Nébéwia Griffete

**Affiliations:** ^1^ Physico‐chimie des Électrolytes et Nanosystèmes Interfaciaux, PHENIX Sorbonne Université CNRS Paris F‐75005 France; ^2^ CNRS Enzyme and Cell Engineering Laboratory Université deTechnologie de Compiègne Rue du Docteur Schweitzer, CS 60319 Compiègne 60203 France; ^3^ Sorbonne Université, CNRS Institut Parisien de Chimie Moléculaire 4 Place Jussieu, CC 229, F‐75252 Paris Cedex 05 France

**Keywords:** molecularly imprinted polymer, photoinduced metal‐free ATRP, poly(divinylbenzene), polymer microsphere, precipitation polymerization, uniform

## Abstract

Herein, a photoinduced method is introduced for the synthesis of highly cross‐linked and uniform polymer microspheres by atom transfer radical polymerization (ATRP) at room temperature and in the absence of stabilizers or surfactants. Uniform particles are obtained at monomer concentrations as high as 10% (by volume), with polymers being exempt from contamination by residual transition metal catalysts, thereby overcoming the two major longstanding problems associated with thermally initiated ATRP‐mediated precipitation polymerization. Moreover, the obtained particles have also immobilized ATRP initiators on their surface, which directly enables the controlled growth of densely grafted polymer layers with adjustable thickness and a well‐defined chemical composition. The method is then employed successfully for the synthesis of molecularly imprinted polymer microspheres.

## Graphical abstract

A new photoinduced method for the synthesis of highly cross‐linked and uniform polymer microspheres by atom transfer radical polymerization at room temperature and in the absence of stabilizers or surfactants has been developed. Particles are obtained at monomer concentrations as high as 10% (by volume), with polymers being exempt from contamination by residual transition metal catalysts.

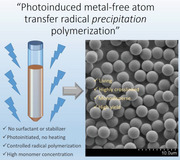

## 1 Introduction

From column chromatography to molecular imprinting, highly crosslinked and uniform polymer microspheres have found many applications in materials science.^[^ ^1^ ^]^ Since 1993, precipitation polymerization (PP) by thermally mediated free‐radical polymerization (FRP) has been a powerful yet easy method to synthesize these particles in the absence of stabilizers or surfactants of any kind.^[^ ^2^ ^]^ Subsequently, the method was used for the preparation of uniform core–shell,^[^ ^3^ ^]^ porous,^[^ ^4^ ^]^ and chloromethylstyrene functionalized ^[^ ^5^ ^]^ particles. Thereafter, photoinitiated PP,^[^ ^6^ ^]^ distillation PP,^[^ ^7^ ^]^ reflux PP,^[^ ^8^ ^]^ and solvothermal PP^[^ ^9^ ^]^ have also been successfully applied to the synthesis of uniform polymer particles. Photoinitiated PP was shown to produce uniform particles at ambient temperature, whereas distillation and solvothermal PP enabled the synthesis of these particles in relatively short times with improved yields.^[^ ^7^, ^9^ ^]^ The use of light for polymer microsphere synthesis has huge potential in materials science and paves the way for the development of milder and safer reaction conditions.^[^ ^10^ ^]^ Compared to its thermal analog, photoinduced synthesis enables the design of safer reaction conditions since it minimizes the risk of pressure‐related explosions and is energy efficient since it enables the reactions to be carried out at ambient temperature and pressure, which are two fundamental principles of green chemistry.^[^ ^11^ ^]^ Inspired by the potential and advantages of light‐induced synthesis in material design, we recently developed “type II photoinitiated PP,” a method that enables the synthesis and in‐situ functionalization of polymer microspheres by UV irradiation.^[^ ^12^ ^]^ Following this, we demonstrated a one‐step synthesis of fluorescent microspheres,^[^ ^13^ ^]^ which proved also adapted for the synthesis of fluorescent hydrogels and nanogels, later on.^[^ ^14^ ^]^

Reversible‐deactivation radical polymerization (RDRP) plays a key role in advanced material design since it enables the synthesis of polymers with well‐defined architectures, predetermined molecular weights, and narrow molecular weight distributions.^[^ ^15^ ^]^ In particular, ATRP has been one of the most powerful RDRP methods since 1995,^[^ ^16^ ^]^ and several complex structures such as star,^[^ ^17^ ^]^ block,^[^ ^18^ ^]^ and graft^[^ ^19^ ^]^ copolymers were prepared by this method. Despite being a versatile method, a major drawback of the initially developed ATRP was the transition metal contamination due to the incomplete removal of the metal catalyst, which was partially solved by the development of new ATRP methods requiring lower amounts of catalysts^[^ ^20^ ^]^ and by the use of resins with immobilized metal catalysts.^[^ ^21^ ^]^ These methods yielded polymers with less metal catalyst contamination and with high levels of chain‐end functionality due to the suppressed side reactions between the growing polymer chains and metal catalysts.^[^ ^22^ ^]^ A major breakthrough appeared in 2014, when a photoinduced and completely transition metal catalyst‐free ATRP, based on the use of an organic photosensitizer for electron transfer, was developed.^[^ ^23^ ^]^

RDRPs have also been combined with PP, for the synthesis of polymer particles.^[^ ^24^ ^]^ Adapting the initiation mechanism of RDRPs to PP enables the synthesis of polymer microspheres with living chain ends. These particles can be used directly in surface‐initiated polymerizations without any modification on the particle surface. The first combined use of RDRPs with PP was reported in 2009 by Zhang and co‐workers, for the synthesis of molecularly imprinted polymers (MIPs) using traditional copper‐based ATRP.^[^ ^25^ ^]^ Although polydisperse, the particles prepared by the new method, atom transfer radical precipitation polymerization (ATRPP), performed better compared to their analogs prepared by free radical precipitation polymerization (FRPP). Following this remarkable work, uniform polymer particles were synthesized by the same group using traditional ATRPP,^[^ ^26^ ^]^ reverse ATRPP,^[^ ^27^ ^]^ and ATRPP conducted at room temperature in different alcohols.^[^ ^28^ ^]^ Despite being a powerful tool to prepare polymer particles in the micron range, a major drawback of the abovementioned ATRPP methods is the transition metal catalyst (very often copper) contamination. To address this problem, more recently, we reported the first combined use of activators regenerated by electron transfer (ARGET)ATRP with precipitation polymerization, enabling the synthesis of uniform particles at very low copper concentrations.^[^ ^29^ ^]^ However, uniform particles could only be obtained at low monomer concentrations and the yield of polymerization was relatively low. Encouraged by its compatibility with acidic monomers, photoinduced ATRP was employed for the synthesis of nano‐size MIPs at room temperature, using methacrylic acid (MAA) as a functional monomer.^[^ ^30^ ^]^ Later on, electrochemically mediated ATRPP was also adapted for the synthesis of MIPs in the nanometer range.^[^ ^31^ ^]^ In addition to catalyst contamination and the necessity of heat for polymerization, previously developed ATRPP methods operate at low monomer concentrations (typically 2% or below) to prevent the aggregation of particles.

Inspired by the need for the production of well‐defined polymer microspheres under energy‐efficient, mild, and safe reaction conditions and by the recent developments in RDRPs, herein, we introduce a light‐induced and completely transition metal catalyst‐free method for the synthesis of micron‐size, highly crosslinked, and uniform spherical polymer particles by combining photoinduced metal‐free ATRP with PP. The particles are synthesized in the absence of stabilizers or surfactants of any type at room temperature by UV irradiation and they possess immobilized initiators on their surface, which enables the controlled growth of polymer layers from their surface without the need for further modification.

## 2 Results and Discussion

**Figure 1:**
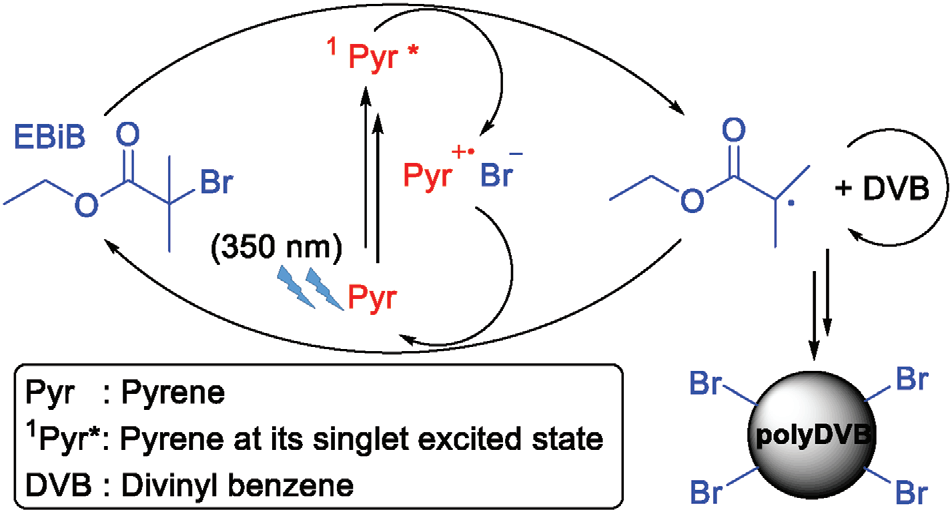
The mechanism of particle formation in photoinduced metal‐free ATRPP.

Photoinduced metal‐free ATRPP is adapted to PP by using ethyl α‐bromoisobutyrate (EBiB) and pyrene, as the initiator/photocatalyst pair. Upon irradiation, the ground state pyrene molecule is excited to its singlet excited state and it transfers an electron to EBiB to form a tertiary radical that initiates polymerization. The simultaneously formed pyrene radical cation receives an electron from the bromide anion and the ground state pyrene is reformed (**Figure** **1**).

PP starts with a homogenous mixture of monomer(s), initiator, and solvent. As the reaction proceeds, the reaction mixture becomes heterogeneous due to the formation and phase separation of the initially formed particles. When polymers are prepared by PP under the FRP mechanism, particles grow by capturing radical oligomers via their unreacted double bonds on their surface.^[^ ^32^ ^]^ On the other hand, particles prepared by RDRPs grow analogues to the surface‐initiated polymerization mechanism. In ATRP, the rate of initiation is fast, the rate of propagation is slow, and termination by radical‐radical coupling is limited. Once the initial nuclei are formed, particles continue to grow similarly to surface‐initiated ATRP, since the vast majority of the initiator molecules are expected to be immobilized on the particles at this stage.

To confirm the ATRP mechanism for particle formation, initial experiments were carried out at different initiator concentrations. For this purpose, divinylbenzene (DVB) concentration was set to 2%, which represents a standard value for PP to obtain uniform particles, and pyrene to DVB mole ratio was gradually increased from 0.5:100 to 8:100, while pyrene to EBiB mole ratio (1:2.4) and other reaction parameters were held constant.

**Figure 2:**
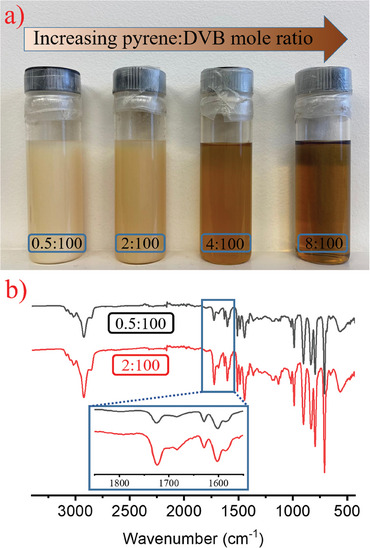
The picture of the reaction mixtures a), and the FTIR spectra of the polyDVB particles b) prepared at different pyrene:DVB mole ratios, after 42 h of irradiation.

**Figure** **2**a shows the picture of the reaction mixtures prepared at different initiator concentrations after 42 h of irradiation: as can be seen from the image, the yield of insoluble particles decreased as the initiator concentration was raised, which is consistent with the concomitant increase of soluble (branched) particles. The gravimetric yield of phase‐separated particles decreased from 6.9 to 4.7%, as the pyrene:DVB mole ratio was raised from 0.5:100 to 2:100. At 4:100 mole ratio, this value was less than 1% and no precipitated particles were obtained at 8:100 ratios, possibly because only soluble and/or branched DVB oligomers are formed at this stage.

Figure 2b shows the FTIR spectra of polyDVB particles prepared at different initiator concentrations. As the initiator concentration is raised, the intensity of the peak at 1724 cm^−1^, which corresponds to the (C═O) stretching for EBiB, becomes more predominant, confirming the presence of higher ATRP initiator moiety per the same amount of polyDVB particles that supports the hypothesis—particles are formed by photoinduced metal‐free ATRP process.

**Figure 3:**
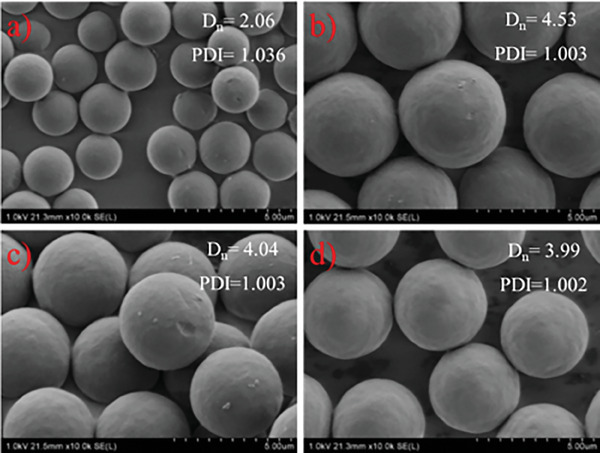
SEM images of the particles were prepared at 2% a), 3% b), 4% c), and 5% d) monomer concentrations (The scale bar corresponds to 5 µm).

The monomer concentration is known to influence particle properties in precipitation polymerization conducted under both FRP and ATRP conditions. It was then expected that both gravimetric yield and particle size should be affected by this parameter. To confirm this, pyrene to DVB mole ratio was set to 1:100 and DVB concentration was raised gradually from 2% to 5%, which are typical values to obtain uniform and spherical particles in PP. Consistently with literature data,^[^ ^7^, ^26^ ^]^ the size of particles and gravimetric yield increased, as the monomer concentration was raised in the feed. Particle size increased from 2.06 to 4.53 µm, as the monomer concentration was raised from 2% to 3%. When the monomer concentration was further increased, its influence on particle size was less noticeable, and even a slight decrease (4.04 and 3.99 µm for 4 and 5%, respectively) was observed in particle size (**Figure** **3**).

Despite being a powerful tool for synthesizing uniform particles in the micron range, a major drawback of PP is the low monomer concentration required to prevent the aggregation of particles, which causes the loss of a high amount of solvent.

**Figure 4:**
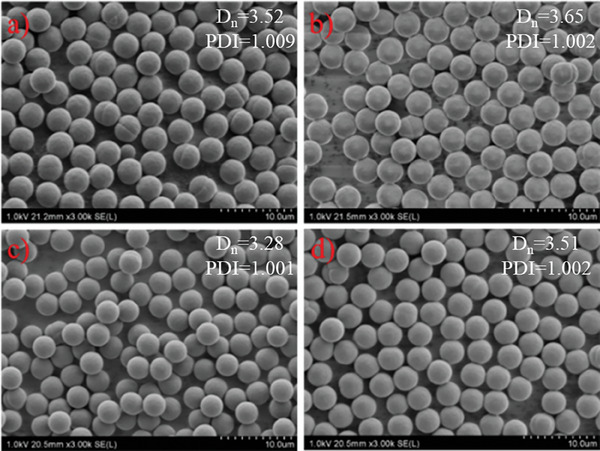
SEM images of the particles were prepared at 7% a), 8% b), 9% c), and 10% d) monomer concentrations.

To address this problem and inspired by the possibility of carrying FRPP at high monomer concentrations when initiated by UV light,^[^ ^6a^ ^]^ we also wanted to investigate the upper limit of monomer concentration for microsphere synthesis. For this purpose, we gradually increased the monomer concentration while keeping other reaction parameters, including monomer to initiator ratio, constant. It was observed that uniform particles were still formed at monomer concentrations as high as 10% (**Figure** **4**). As the monomer concentration was raised from 7 to 8% (v/v), the particle size increased from 3.52 to 3.65 µm and as the monomer concentration was raised from 9 to 10% (v/v), the particle size increased from 3.28 to 3.51 µm. Tests at 7–8% and 9–10% monomer concentrations were conducted separately and the slightly bigger size observed at 7% compared to 9% may be related to the slightly different position of the vials with respect to the light source. It is also important to notice that although the particle size increased as the monomer concentration was raised, the increase in particle size was less noticeable compared to the size increase when the monomer concentration is raised from 2% to 5%. The volume of a sphere is 4/3πr^3^, meaning that the volume of a sphere having 2r radius will be eight times bigger than a sphere having r radius. Therefore, to double the radius of a polyDVB particle, ≈7 times more monomer should be consumed. Given the fact that the monomer concentration is continuously decreasing in the reaction medium, the slow increase in the particle size as the reaction proceeds is quite meaningful.

**Figure 5:**
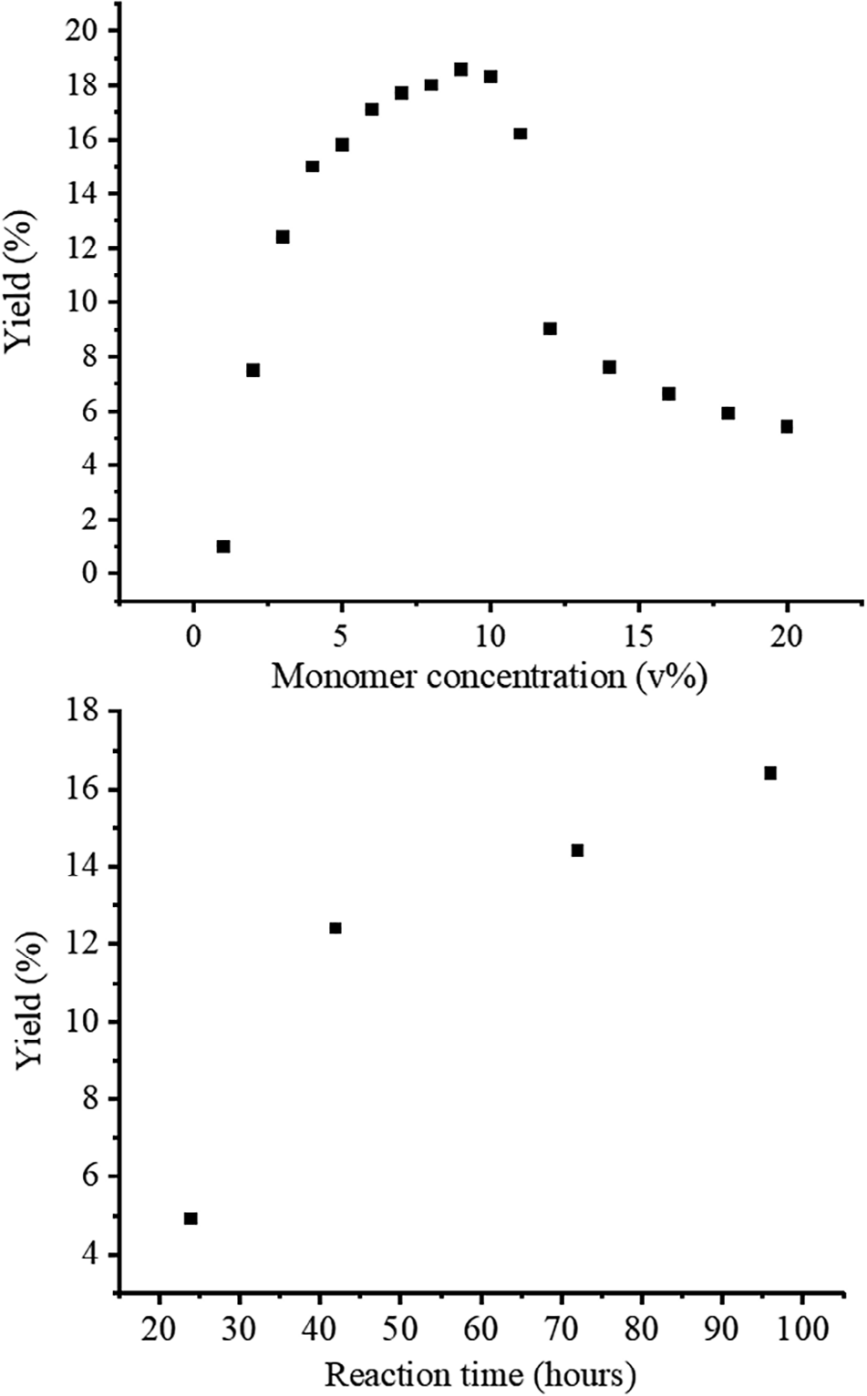
The effect of monomer concentration a) and reaction time b) on the yield of polymerization.

To test the limit of monomer concentration to obtain uniform particles, we continued to raise the monomer concentration in the feed. As the monomer concentration is further raised to 11% and 12%, still spherical and uniform particles were obtained; however, secondary nucleation started at this stage (Figure S1, Supporting Information). At 14% monomer concentration, cauliflower‐like structures rather than spheres started to form, probably due to the aggregation of the particles at the nucleation and particle growth stages. Even at 20% monomer concentration, though not uniform, discrete and micron‐size particles were obtained, which may still be useful for special applications. **Figure** **5**a summarizes the data obtained for gravimetric yield versus monomer concentration. The yield of polymer particles increased until 9% monomer concentration, where it reached 18.6%.

At 10% monomer concentration, the yield was 18.3% and after this point, an exponential decrease, probably due to the inefficient irradiation of the reaction mixture resulting from the formation of the suspension, was observed. At 12% monomer concentration, the majority of the particles started to stick on the walls of the borosilicate reaction tube and the same phenomenon was observed until 20% monomer concentration, where the yield was 5.4%.

Next, we investigated the influence of reaction time on the gravimetric yield, and the reactions were carried out at 3% monomer concentration. As the reaction time is raised from 24 to 48 h, the yield of polymerization increased from 4.9 to 12.4% (Figure 5b). The increase in yield was less noticeable starting from this point and it was 14.4 and 16.4% for 72 and 96 h, respectively. The decrease in the rate of gravimetric yield increase is possibly related to light‐screen through the reaction mixture, which is due to the formation of the insoluble particles, as mentioned above. These results imply that the yield of polymerization could further be increased if the reaction would have been carried out for more than 42 h, albeit to a lesser extent. The SEM images of the particles obtained at 24, 72, and 96 h are presented in Figure S2 (Supporting Information).

Swelling ability and pore characteristics of the polymer particles are strongly dependent on their crosslinking density, which should be tuned depending on the application. Therefore, to show the applicability of the developed strategy on the synthesis of polyDVB particles with different crosslink ratios, the effect of DVB composition in the feed on particle properties, was also studied.

Commercial technical grade DVB comprises 55 or 80% DVB and the rest is ethylvinylbenzene, both of which are mixtures of meta and para isomers. DVB compositions between these values (61.3, 67.5, and 73.8%) were obtained by simply mixing the two commercially available grades in 1:1 and 1:2 (v/v) ratios.

**Figure 6:**
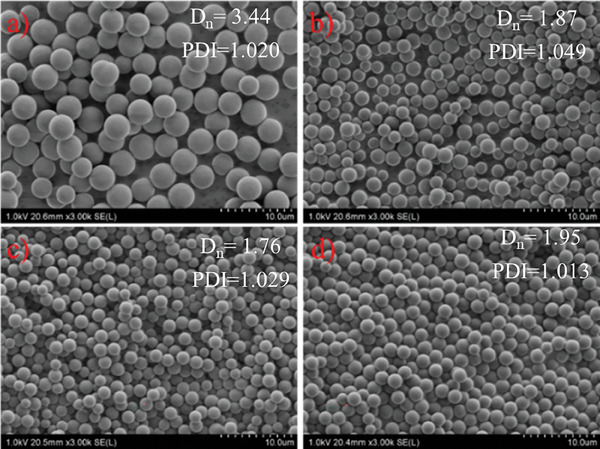
SEM images of polyDVB particles were prepared with 55.0% a), 61.3% b), 67.5% c), and 73.8% d) DVB.

**Figure 7:**
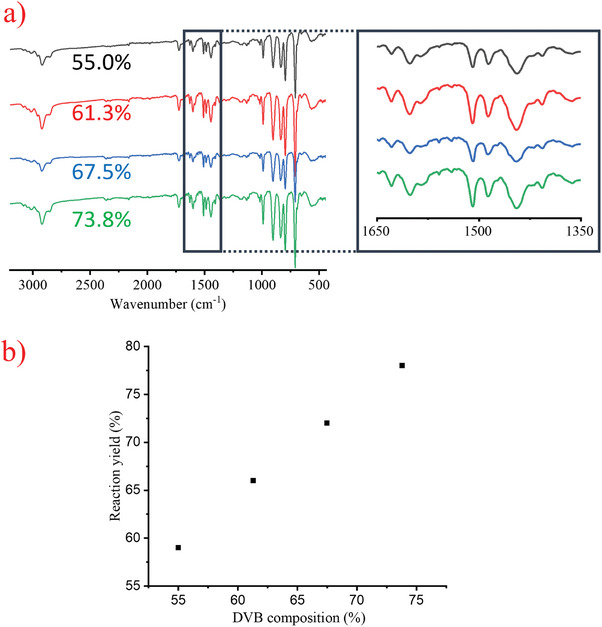
FTIR spectra a) and the yield versus DVB composition graph b) of polyDVB particles prepared with 55.0%, 61.3%, 67.5%, and 73.8% DVB.

**Figure** **6** shows the SEM images of polyDVB particles prepared at these ratios. As can be seen, uniform particles were obtained at each DVB composition and the particle size decreased as the DVB composition is raised in the feed (3.44, 1.87, and 1.76 µm for 55.0, 61.3, and 67.5% DVB, respectively). Interestingly, a slight increase in particle size (1.95 µm) was observed as the DVB composition was set to 73.8%. When DVB composition is gradually raised from 55 to 73.8% with 6.25% increment each time, the amount of isolated product increased from 59 to 78 mg, which corresponds to 7.2% and 9.5% yield, respectively, with a very good correlation (6 mg increase in the amount of isolated products almost every time: 59, 66, 72, and 78 mg of products were isolated at 55.0, 61.3, 67.5, and 73.8% DVB compositions, respectively) (**Figure** **7**b). The increase seems logical since reaction mixtures when prepared with less crosslinker amount, should include a more soluble fraction at the end of the polymerization, which is then washed off during the filtration step. As the DVB composition is raised in the feed, the amount of unreacted double bonds in the polymer network also increases, which is confirmed by the increase in the intensity of the peak at 1629 cm^−1^ that corresponds to the C═C stretching vibration of DVB, in the FTIR spectra (Figure 7a).

**Figure 8:**
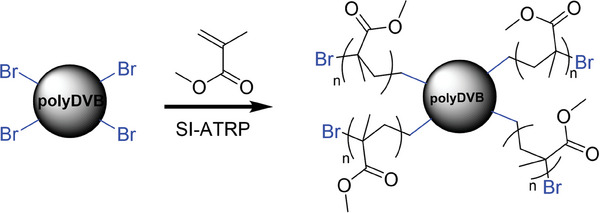
Schematic representation of the growing polymer brushes from the particle surface via ATRP.

Controlled growth of polymer layers from surfaces via RDRPs enables the synthesis of densely grafted polymer layers with adjustable thickness and well‐defined chemical composition. Conventionally, it requires sequential particle synthesis and immobilization of initiator molecules onto the particles, which is followed by surface‐initiated polymerization.^[^ ^33^ ^]^ Combining RDRPs with PP has the advantage of obtaining polymer particles with already immobilized initiator molecules on their surface in a single step, enabling their direct use in grafting from experiments without any further modification. Photoinduced metal‐free ATRPP is no exception; therefore, the particles prepared by this method should also possess initiator molecules on their surface. To test this and to further confirm that photoinduced metal‐free ATRPP is the dominant process for particle formation, microspheres were utilized in grafting from experiments without any modification on their surface to immobilize initiators. For this purpose, 50 mg of dry polyDVB particles were re‐dispersed in 4 mL methyl methacrylate (MMA) and experiments were carried out in the absence of EBiB to ensure grafting is initiated from the surface of the particles (**Figure** **8**).

**Figure 9:**
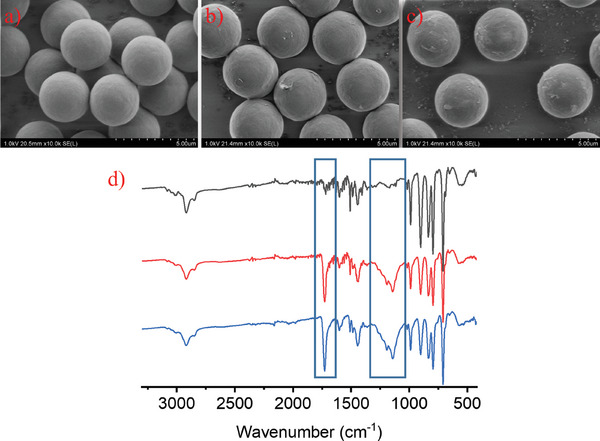
SEM images of the polymer microspheres before a), and after 24 h b), and 48 h c) of grafting. FTIR spectra of the polymer microspheres before (top), and after 24 h (middle), and 48 h (bottom) of grafting d).

For grafting experiments, particles prepared at 9% monomer concentration were used as seed particles due to their uniformity (**Figure** **9**a) and MMA was used as the grafting monomer since it can be easily detectable by FTIR due to its carbonyl group. To avoid light‐screening from particles, we rather preferred at this stage a conventional metal‐catalyzed thermal ATRP. After 24 h of grafting at 60 °C, the surface of the particles was no longer smooth and the number average particle diameter increased from 3.28 to 3.45 µm (Figure 9b). When the grafting from experiments was further carried out for an additional 24 h at 70 °C, the cloudy grafted polyMMA layer was more noticeable; however, no practical change in size was observed (3.46 µm) (Figure 9c). The formation of the polyMMA shell was further supported by FTIR (Figure 9d). After 24 h of grafting, a strong peak appears at 1727 cm^−1^ due to the carbonyl stretching and a broad signal appears from 1040 to 1300 cm^−1^, resulting from C─O─C stretching vibration for grafted polyMMA brushes. These signals became more intense when the polymerization was carried out for a further 24 h, which was consistent with the growth of the shell layer, further demonstrating the success of grafting from experiments.

Thermogravimetric analysis under nitrogen (Figure S7, Supporting Information) shows that the residual mass percentage changed from 16.49% (before grafting) to 17.17% (after 24 h of grafting) and 19.46% (after 48 h). Moreover, two different degradation temperatures appear after 24 h (393 and 429 °C) and 48 h (399 and 431 °C) MMA polymerization when only one degradation temperature is visible before the growth of the second polymer layer (437 °C). These results indicate that the polymer has successfully been grafted onto the particles.

MIPs are synthetic receptors that can bind preselected target molecules selectively.^[^ ^34^ ^]^ Despite the presence of other forms, MIP microspheres are of unique importance since they can be packed into columns, enabling their direct use for chromatography as HPLC packing materials. However, a major drawback of MIP microsphere synthesis is the requirement of heat for polymerization, which weakens the complexation forces between template and functional monomer in most cases. In addition, traditional copper‐based ATRP is known to be incompatible with acidic monomers, and transition metal catalysts may interfere with the templating process, making ATRP unsuitable for its use to prepare MIPs. When employed for MIP synthesis, photoinduced metal‐free ATRP has been shown to be compatible with MAA, a functional monomer commonly used for imprinting.^[^ ^30^ ^]^ In addition, reactions can be carried out at room temperature, which makes the method compatible with heat‐sensitive compounds, overcoming another limitation for MIP synthesis. Therefore, in the next step, the developed method, photoinduced metal‐free ATRPP, was employed for the synthesis of R‐PhEtOH imprinted MIP microspheres, and equilibrium binding experiments were carried out in order to assess the imprinted polymers' selectivity. For this purpose, four different alcohols were selected, whose chemical structures are presented in Figure S3 (Supporting Information).

**Figure 10:**
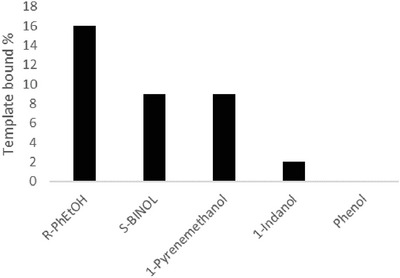
Binding results of the R‐PhEtOH imprinted polymers toward other alcohol‐bearing molecules.

The MIPs showed the highest amount of binding for R‐PhEtOH (16%), and the closest and highest values were obtained for S‐BINOL and 1‐ pyrenemethanol, both of which were 9%. MIPs bound 1‐indanol only 2%, and no binding was observed for phenol (**Figure** **10**).

## 3 Conclusion

Motivated by the necessity to prepare well‐defined polymer particles in milder and safer reaction conditions, we introduced a metal‐free photoinduced ATRP method for the synthesis of highly cross‐linked uniform polymer microspheres at room temperature and in the absence of stabilizers or surfactants of any kind. This new method is called “photoinitiated metal‐free atom transfer radical precipitation polymerization” and uniform particles from 2.06 to 4.53 µm can be obtained at high monomer concentrations (e.g., 10%) with good yield (e.g.,18.6%) even after 42 h of irradiation. It is also demonstrated that the resulting particles can be directly used in surface‐initiated polymerization to grow polymer brushes from the surface without the need for further modification, due to the living nature of the microspheres. This method is of general applicability as the irradiation wavelength can in principle be adjusted simply by replacing pyrene with other photosensitizers, which allows using a broad range of the electromagnetic spectrum for particle synthesis. In addition, the method developed here is expected to be compatible with acidic monomers due to the inherent nature of the photo ATRP process, expanding the use of these materials in areas such as MIP‐based solid phase extraction column chromatography and paving the way for the development of a new class of well‐defined materials under energy‐efficient conditions. Our results clearly demonstrate that photoinduced metal‐free ATRPP can be employed for the synthesis of MIP microspheres, paving the way for a new and transition metal catalyst‐free ATRP method at room temperature, which enables the use of MAA as a functional monomer to prepare MIPs.

## Conflict of Interest

The authors declare no conflict of interest.

## Supporting information



Supporting Information

## Data Availability

The data that support the findings of this study are available from the corresponding author upon reasonable request.
