# Supplementary material for: Uniform Polymer Microspheres by Photoinduced Metal‐Free Atom Transfer Radical Precipitation Polymerization
Source: Macromol Rapid Commun. 2024 Nov 5;46(1):2400502. doi: 10.1002/marc.202400502 (PMC11713857; doi:10.1002/marc.202400502)
Supplement: Supplementary file 1 — Supporting Information [file MARC-46-2400502-s001.docx]

Supporting Information
©Wiley-VCH 2021
69451 Weinheim, Germany

**Uniform Polymer Microspheres by Photoinduced Metal-Free Atom Transfer Radical Precipitation Polymerization**

Tugrul Cem Bicak,*^[a]^ Huiyin Liu,^[a]^ Karsten Haupt, ^[b]^ Carlo Gonzato, ^[b]^ Jérôme Fresnais,^[a]^ Christine Ménager,^[a]^ Louis Fensterbank,^[b]^ Cyril Ollivier, ^[a]^ Nébéwia Griffete*^[a]^

[a] Dr. T. C. Bicak, H. Liu, Dr. J. Fresnais, Prof. C. Ménager, Dr. N. Griffete
Physico-chimie des Électrolytes et Nanosystèmes Interfaciaux, PHENIX,

Sorbonne Université CNRS Paris F-75005, France

E-mail: tugrul.bicak@sorbonne-universite.fr, nebewia.griffete@sorbonne-universite.fr

[b] Prof. K. Haupt, Dr. C. Gonzato

CNRS Enzyme and Cell Engineering Laboratory, Université deTechnologie de Compiègne

Rue du Docteur Schweitzer, CS 60319,60203 Compiègne Cedex, France

[c] Prof. L. Fensterbank, Prof. C. Ollivier

Sorbonne Université, CNRS, Institut Parisien de Chimie Moléculaire

4 Place Jussieu, CC 229, F-75252 Paris Cedex 05, France

**Abstract:** Reversible deactivation radical polymerizations have a key role in advanced materials design and the use of light for polymer synthesis paves the way for the development of milder and safer reaction procedures with increased energy efficiency, which are the two fundamental principles of green chemistry. Herein, we introduce a photoinduced method for highly crosslinked and monodisperse microsphere synthesis by atom transfer radical polymerization (ATRP) at room temperature in the absence of stabilizers or surfactants. Monodisperse microspheres are obtained at monomer concentrations as high as 10% (by volume) and the particles are completely free from residual transition metal complex catalyst contamination, overcoming the two major problems associated with the traditional thermally initiated and ATRP methods for particle synthesis. Moreover, particles obtained by this method also have immobilised ATRP initiators on their surface, enabling the controlled growth of densely grafted polymer layers with adjustable thickness and well-defined chemical composition without the need for a further modification.

Experimental Procedures

Materials: Divinylbenzene, consisting of 80% divinylbenzene isomers (DVB-80, technical grade, Aldrich) and consisting of 55% divinylbenzene isomers (DVB-55, technical grade, Aldrich), were purified by passing through a basic alumina column to remove inhibitor. Copper(I) bromide (CuBr, 99%, Aldrich), acetonitrile (ACN, 99%, Aldrich), N,N,N′,N′′,N′′-pentamethyldiethylenetriamine (PMDETA, 99%, Aldrich), pyrene (), methyl methacrylate (MMA, 99%, Aldrich), α-bromoisobutyryl bromide (98%, Aldrich), acetone (99%, Aldrich), were used as received.

Methods: Photopolymerisations were carried out at room temperature on a low profile roller operating approximately at 10 rpm and irradiated from a UV light source (VL-215.L 2x15W – 365 nm tube UV lamb, Fischer Bioblock Scientific), which is placed approximately 3 cm above the reaction tube, for 42 h. Fourier Transform Infra-Red (FT-IR). Infrared spectra were obtained on a Nikolet iS20 spectrometer. Spectra were obtained at regular time intervals in the region of 4000 – 400 cm–1 at a resolution of 4 cm–1 and analysed using OMNIC software. Scanning electron microscopy (SEM) images were acquired using a Hitachi SU-70, Schottky gun type, without coating. Image analyses of the SEM micrographs were performed using Image J47 software, on a population of 100 microspheres if enough particles were present on the images. Otherwise, the number of particles used in calculations for each sample is presented in Table S1. The following equations were used to determine the particle sizing characteristics:

$U=\frac{D_{w}}{D_{n}}$; $D_{n}=\sum_{i=1}^{k} \left( n_{i}D_{i} \right)$ / $\sum_{i=1}^{k} \left( n_{i} \right)$; $D_{w}=\sum_{i=1}^{k} \left( n_{i}{D_{i}}^{4} \right)$ / $\sum_{i=1}^{k} \left( n_{i}{D_{i}}^{3} \right)$

where U is the polydispersity index, Dn is the number-average diameter, Dw is the weight-average diameter, N is the total number of the measured particles, and Di is the particle diameters of the microspheres.[10, 16] Polymers were isolated by filtration using a hydrophilic polyvinylidiene fluoride (PVDF) membrane filter discs with 0.22 µm pore size (Durapore, Sigma).

Experimental:

*Preparation of poly(DVB) microspheres by photoinduced metal-free ATRPP:* A typical polymerization procedure is as follows (Table S1, entry 13, sample code: T7-17): To a Pyrex glass tube was added DVB-80 (2.7 mL, 18.96 mmol), pyrene (39 mg, 0.193 mmol), and ACN (27.3 mL), and the mixture was ultrasonicated for few seconds until a clear solution was obtained. Then, the mixture was sparged with nitrogen gas for 10 minutes prior to adding EBiB (69 µL, 0.470 mmol) and sealing of the reaction vessel. The sealed tube was then placed into a low profile roller operating at ~10 rpm and irradiated from a light source that emits light at 365 nm for 42 hours. The polymer microspheres were isolated from the reaction media by vacuum filtration and washed with acetone (~250 mL). Finally, the product was dried overnight in an oven (60 °C) to constant mass (459 mg, 18.6% yield). Reaction mixtures, which were colorless and transparent initially, turned into yellow-brown milky suspensions in the end of polymerizations. The room temperature at the time when the reactions were carried out was roughly between 20 to 25°C. However, the actual reaction temperature might have been slightly above this range due to the overheating of the light bulbs which were placed ~3 cm above the light source.

*Grafting of polymer brushes from polymer microspheres:* Polymer microspheres (50 mg) were added to a 25 mL round-bottomed flask and dispersed in MMA (4 mL). After purging the suspension with nitrogen for 5 min., CuBr (16 mg, 0.11 mmol) and PMDETA (66 μL, 0.38 mmol) were added sequentially and the mixture was deoxigenated for a further 3 min. The flask was then sealed under nitrogen and transferred to an oil bath and the reaction mixture was stirred at 400 rpm for 24 h at 60 °C. Then, the temperature was set to 70 °C and the reaction was continued for a further 24 h. Polymer particles, which were sampled at 24 h and 48 h, were then collected by vacuum filtration and washed with acetone (~250 mL) before drying overnight in vacuo (50 mbar) at 60 °C.

*Synthesis of R-PhEtOH imprinted polymer microspheres*: The R-PhEtOH imprinted polymer microspheres are prepared simply by including the template (R-PhEtOH) and the functional monomer (MAA) during the preparation of particles. To a Pyrex glass tube DVB-80 (1.2 mL, 8.43 mmol), pyrene (16.5 mg, 0.082 mmol), MAA (114 µL, 1.35 mmol), R-PhEtOH (42 µL, 0.34 mmol), and ACN (28.8 mL), were added, and the mixture was ultrasonicated for few seconds until a clear solution was obtained. Then, the mixture was sparged with nitrogen gas for 10 minutes prior to adding EBiB (31 µL, 0.211 mmol) and sealing of the reaction vessel. The sealed tube was then placed into a low profile roller operating at ~10 rpm and irradiated from a light source that emits light at 365 nm for 42 hours. The polymer microspheres were isolated from the reaction media by vacuum filtration and washed with acetone (~150 mL), (10% acetic acid in MeOH). Finally, the product was dried overnight in an oven (60 °C) to constant mass (459 mg, 18.6% yield).

*Equilibrium binding experiments.* For each binding experiment, 8 mg MIP was weighed and suspended in 1 ml solution (heptane/IPA 95/5) containing 1 mM template. All binding experiments were carried out in 10 mL glass vials for 24 hours. After 24 hours of shaking on an orbital shaker, the MIPs were separated from the medium by a disk filter with a 0.2 µm pore size and the concentration of the template in the supernatant was analysed by HPLC (Column: Kromasil, flow rate: 0.5 ml/min, mobile phase: heptane/IPA 95/5).

Results and Discussion

**Table S1.** Summary of the particle characteristics obtained at different reaction conditions

| **ENTRY** | **CODE** | **Monomer conc. (v%)** | **Dn** | **Dw** | **PDI** | **# of particles ^a^** | **YIELD (%)^b^** |
| --- | --- | --- | --- | --- | --- | --- | --- |
| 1 | T7-5 | 2 | 2.06 | 2.13 | 1.036 | 100 | 7.5 |
| 2 | T7-6 | 3 | 4.53 | 4.54 | 1.003 | 49 | 12.4 |
| 3 | T7-7 | 4 | 4.04 | 4.05 | 1.003 | 57 | 15.0 |
| 4 | T7-8 | 5 | 3.99 | 3.99 | 1.002 | 59 | 15.8 |
| 5 | T7-9 | 1 | 2.36 | 3.39 | 1.438 | 100 | <1 |
| 6 | T7-10 | 6 |  |  |  |  | 17.1 |
| 7 | T7-11 | 7 | 3.52 | 3.55 | 1.009 | 95 | 17.7 |
| 8 | T7-12 | 8 | 3.65 | 3.65 | 1.002 | 89 | 18.0 |
| 9 | T7-13 | 2, [pyr:DVB]= [0.5:100]^c^ |  |  |  |  | 6.9 |
| 10 | T7-14 | 2, [pyr:DVB]= [2:100] |  |  |  |  | 4.7 |
| 11 | T7-15 | 2, [pyr:DVB]= [4:100] |  |  |  |  | <1 |
| 12 | T7-16 | 2, [pyr:DVB]= [8:100] | - | - | - | - | - |
| 13 | T7-17 | 9 | 3.28 | 3.29 | 1.001 | 100 | 18.6 |
| 14 | T7-18 | 10 | 3.51 | 3.52 | 1.002 | 89 | 18.3 |
| 15 | T7-19 | 11 |  |  |  |  | 16.2 |
| 16 | T7-20 | 12 |  |  |  |  | 9.0 |
| 17 | T7-21 | 14 |  |  |  |  | 7.6 |
| 18 | T7-22 | 16 |  |  |  |  | 6.6 |
| 19 | T7-23 | 18 |  |  |  |  | 5.9 |
| 20 | T7-24 | 20 |  |  |  |  | 5.4 |
| 21 | T7-25 | 3-(DVB 55) | 3.44 | 3.51 | 1.020 | 92 | 7.2 |
| 22 | T7-26 | 3-(DVB 61.3) | 1.87 | 1.95 | 1.049 | 100 | 8.0 |
| 23 | T7-27 | 3-(DVB 67.5) | 1.76 | 1.81 | 1.029 | 100 | 8.8 |
| 24 | T7-28 | 3-(DVB 73.8) | 1.95 | 1.98 | 1.013 | 100 | 9.6 |
|  | T7-17-1 |  | 3.45 | 3.46 | 1.003 | 52 |  |
|  | T7-17-2 |  | 3.46 | 3.46 | 1.001 | 74 |  |

^a^ The number of particles counted on the SEM images for calculation. ^b^ obtained gravimetrically. ^c^[pyr:DVB] stands for pyrene to DVB mole ratio.


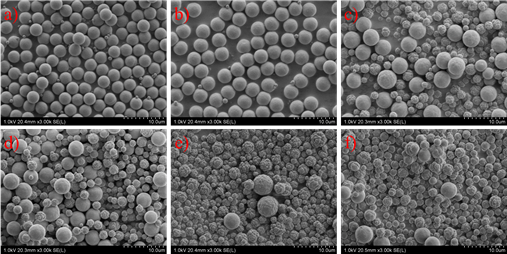


**Figure S1.** SEM images of the particles prepared at 11% (a), 12% (b), 14% (c), 16% (d), 18% (e), and 20% (f) monomer concentrations.




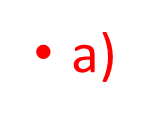

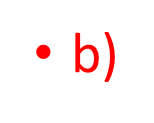

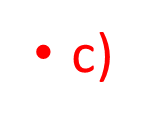







**Figure S2.** SEM images of the particles obtained after 24 (a), 72 (b), and 96 h (c) of polymerization.


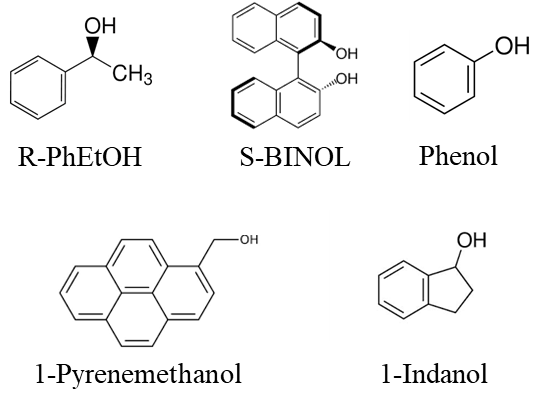


**Figure S3.** Chemical structures of the molecules, which are used in competitive binding experiments.

**Figure S4.** Raw FTIR data for polyDVB particles prepared at [0.5:100] (up) and [2:100] (down) pyrene:DVB mole ratios.

**Figure S5.** Raw FTIR data for polyDVB particles prepared with 55.0%, 61.3%, 67.5%, and 73.8% DVB

**Figure S6.** Raw FTIR data of the polymer microspheres before (top), and after 24 h (middle), and 48 h (bottom) of grafting

**
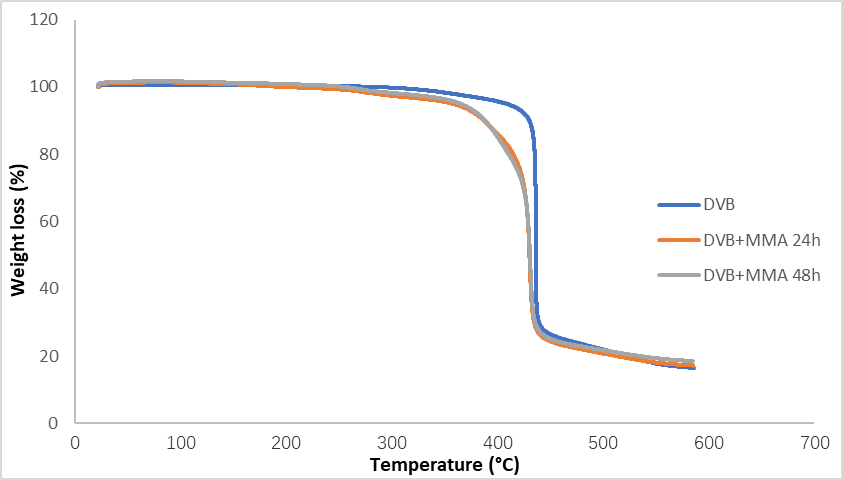
**

**
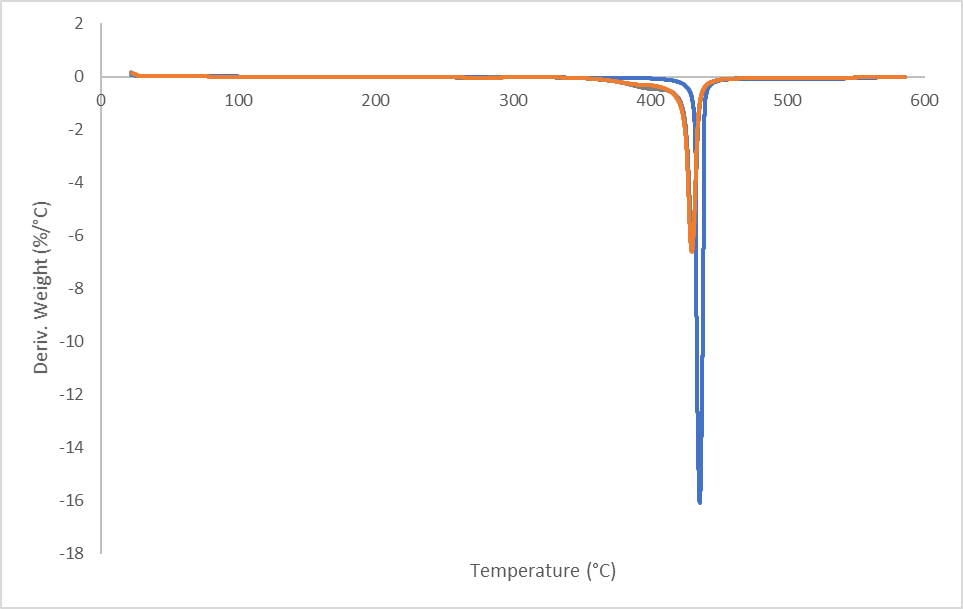
**

**Figure S7.** Thermogravimetric analysis of the polymer microspheres before (blue), and after 24 h (orange), and 48 h (grey) of grafting.

**
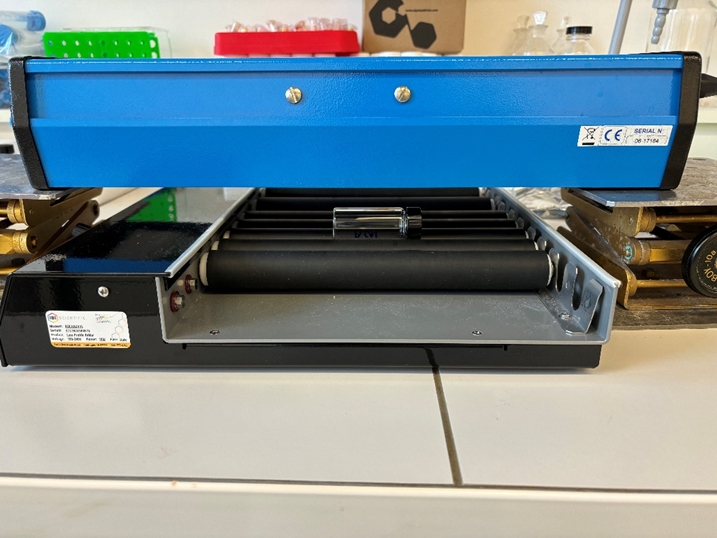
**

**Figure S8.** The photograph of the polymerisation set-up.
